# Failure patterns of locoregional recurrence after reducing target volumes in patients with nasopharyngeal carcinoma receiving adaptive replanning during intensity-modulated radiotherapy: a single-center experience in China

**DOI:** 10.1186/s13014-023-02373-7

**Published:** 2023-11-16

**Authors:** Xiate Zhou, Jian Zhu, Chao Zhou, Wei Wang, Weijun Ding, Meng Chen, Kuifei Chen, Shuling Li, Xiaofeng Chen, Haihua Yang

**Affiliations:** 1 Department of Radiation Oncology, Taizhou Hospital of Zhejiang Province Affiliated to Wenzhou Medical University, Taizhou, 317000 Zhejiang Province China; 2 Department of Radiation Oncology, Enze Hospital, Taizhou Enze Medical Center (Group), Taizhou, 317000 Zhejiang Province China; 3 Key Laboratory of Radiation Oncology of Taizhou, Radiation Oncology Institute of Enze Medical Health Academy, Department of Radiation Oncology, Taizhou Hospital Affiliated to Wenzhou Medical University, Taizhou, 317000 Zhejiang Province China; 4 School of Medicine, Shaoxing University, Shaoxing City, 312000 Zhejiang Province China; 5 Department of Radiation Oncology, Indiana University School of Medicine, Indianapolis, IN 46202 USA

**Keywords:** Nasopharyngeal carcinoma (NPC), Intensity-modulated radiation therapy (IMRT), Replanning, Failure patterns, Target delineation

## Abstract

**Background:**

Previous researches have demonstrated that adaptive replanning during intensity-modulated radiation therapy (IMRT) could enhance the prognosis of patients with nasopharyngeal carcinoma (NPC). However, the delineation of replanning target volumes remains unclear. This study aimed to evaluate the feasibility of reducing target volumes through adaptive replanning during IMRT by analyzing long-term survival outcomes and failure patterns of locoregional recurrence in NPC.

**Methods:**

This study enrolled consecutive NPC patients who received IMRT at our hospital between August 2011 and April 2018. Patients with initially diagnosed, histologically verified, non-metastatic nasopharyngeal cancer were eligible for participation in this study. The location and extent of locoregional recurrences were transferred to pretreatment planning computed tomography for dosimetry analysis.

**Results:**

Among 274 patients, 100 (36.5%) received IMRT without replanning and 174 (63.5%) received IMRT with replanning. Five-year rates of locoregional recurrence-free survival (LRFS) were 90.1% (95%CI, 84.8% to 95.4%) and 80.8% (95%CI, 72.0% to 89.6%) for patients with and without replanning, P = 0.045. There were 17 locoregional recurrences in 15 patients among patients with replanning, of which 1 (5.9%) was out-field and 16 (94.1%) were in-field. Among patients without replanning, 19 patients developed locoregional recurrences, of which 1 (5.3%) was out-field, 2 (10.5%) were marginal, and 16 (84.2%) were in-field.

**Conclusions:**

In-field failure inside the high dose area was the most common locoregional recurrent pattern for non-metastatic NPC. Adapting the target volumes and modifying the radiation dose prescribed to the area of tumor reduction during IMRT was feasible and would not cause additional recurrence in the shrunken area.

**Supplementary Information:**

The online version contains supplementary material available at 10.1186/s13014-023-02373-7.

## Introduction

Nasopharyngeal carcinoma (NPC) is a type of epithelial cancer with an asymmetrical geographic distribution that is prominent in Southern China and Southeast Asia [1, 2]. Intensity-modulated radiation therapy (IMRT) has replaced conventional radiation therapy (CRT) as the benchmark for the treatment of NPC in recent decades, due to its superiority in dose distribution with more accurate dose homogeneity around targets and better sparing of surrounding normal structures [3]. The encouraging advantages in locoregional recurrence-free survival (LRFS), overall survival (OS), side effects, and quality of life (QoL) have been reported consistently in a number of studies [4, 5]. However, the contour changes caused by tumor shrinkage and spatial variability were observed very frequently during radiotherapy (RT) [6]. These changes typically result in a significant difference between planned and delivered doses [6, 7].

Adaptive radiotherapy (ART) is an image feedback control approach that modifies the treatment plan to account for anatomical changes during RT [8]. It has long been recommended especially in NPC because of its advantages to ensure accurate dose for target volumes and safe dose for essential normal structures [9]. However, the delineation of replanning target volumes is still confusing. Should the area of tumor reduction be described as gross target volumes (GTVs) or clinical target volumes (CTVs) during IMRT with replanning? Although a few investigations have observed that adapting the target volumes relying on the re-simulated computed tomography (CT) scans could achieve satisfactory LRFS, alleviate the side effects, and improve QoL [10, 11], reports about the failure patterns of locoregional recurrence are lacking. Due to the highly infiltrative behavior of NPC, concerns about some microscopic tumor cells that can thrive around the area of tumor shrinkage have also been raised since they can lead to an increase in locoregional failures. In this work, we evaluated our long-term follow-up findings, involving survival outcomes and patterns of failure, to give additional support for the target volumes description of adaptive replanning in NPC.

## Methods

### Patients

**Fig. 1:**
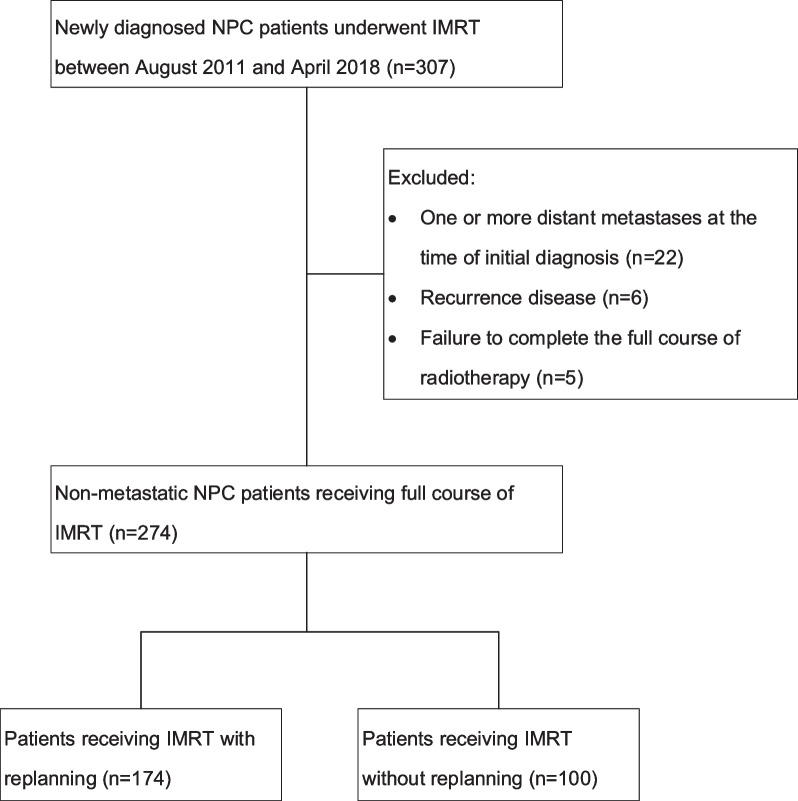
The diagram for the study

Our study enrolled consecutive patients diagnosed with NPC by histology and who had received IMRT at our medical center between August 2011 and April 2018. In total, 307 patients were identified, and 274 patients took part in the research. One hundred and seventy-nine patients who were treated between August 2011 and December 2015 were part of our previous study [12]. Patients with at least one metastatic disease at the time of first diagnosis (n = 22), recurrent disease (n = 6), and failure to accomplish the full course of IMRT (n = 5) were eliminated from the study. Figure 1 depicts the diagram for the research. Our medical center's institutional review board authorized this study.

### Chemotherapy

Patients diagnosed as stage I NPC received only IMRT, while those with stage II-IVa received concurrent chemotherapy. At the beginning of the study, adjuvant chemotherapy (AC) was routinely applied for patients with stage II-IVa NPC. From July 2016, induction chemotherapy (IC) gradually replaced AC as the conventional therapy strategy for patients with locally advanced NPC. There were two main AC and IC regimens, paclitaxel 135 mg/m^2^ on day 1, combined with cisplatin 25–30 mg/m^2^ throughout days 1–3 or docetaxel 75 mg/m^2^ on day 1, combined with cisplatin 25–30 mg/m^2^ throughout days 1–3, with intravenous infusion and 2–3 courses every 3 weeks. Two main concurrent chemotherapy regimens were also used, one was paclitaxel 35 mg/m^2^ as we previously reported [13], another was cisplatin 25–30 mg/m^2^, with infusions lasting three hours and five-six sessions administered weekly in conjunction with IMRT.

### Intensity-modulated radiation therapy

All patients performed simulation CT scan from the skull vertex to 2 cm below the clavicles with a slice thickness of 2.5 mm within 2 days prior to IMRT. Eclipse (Version 10.0, Varian Medical Systems, Palo Alto, CA 94304, USA) and Pinnacle (Philips Radiation Oncology Systems, Milpitas, CA) treatment planning system were used to develop IMRT plans.

The GTVs of nasopharynx (GTVnx) and neck lymph nodes (GTVnd) were defined on the basis of magnetic resonance imaging (MRI) or CT scans, endoscopy, and clinical findings. For patients who received AC, the post chemotherapy volume of the primary lesion and the lymph nodes was used for GTVs delineation. The planning gross target volumes (PGTVs) including PGTVnx and PGTVnd were described as an additional 3 mm margin from GTVnx and GTVnd, respectively, to account for the errors of set-up and movement of the internal organ. The CTV1 was identified as the high-risk regions of tumor invasion and nodal involvement. The CTV2 was identified as the low-risk regions. The planning target volumes (PTVs) including PTV1 and PTV2 were described as an additional 3 mm margin from CTV1 and CTV2, respectively. The simultaneous integrated boost (SIB) technique was used, and the IMRT prescription was 70 to 76 Gy at 2.12 to 2.3 Gy to PGTVnx, 66 to 70 Gy at 2.0 to 2.12 Gy to PGTVnd, 60 to 66 Gy at 1.8 to 2.0 Gy to PTV1, and 56 to 60 Gy at 1.7 to 1.8 Gy to PTV2, in 33 fractions. For the purposes of optimization and evaluation, the RTOG 0225 protocol from the Radiation Therapy Oncology Group was employed as a standard constraint set [14].

### Adaptive replanning

Prior to receiving treatment, patients were instructed to undergo adaptive replanning, with the detailed procedure, potential benefits (improved locoregional control, reduced toxicity), and the downsides (added expenses, prolonged treatment times) were particularly explained and the patients consented. Ultimately, patients who declined a second simulation CT scan during the course of IMRT were given IMRT without adaptive replanning, while other patients had IMRT with replanning. The re-simulation CT scan and replanning were carried out at the 15th and/or the 25th fraction of IMRT as we previously reported [15]. The replanning GTVs was described as residual shrunken tumor and positive lymph nodes. The replanning CTVs were outlined with the same extended margin from the corresponding GTVs and its involved regions were described as identical to the original plans with anatomical modifications. Tumor regression area was included in the replanning CTV1. The replanning PGTVs and PTVs were defined as 3 mm extensions from the replanning GTVs and CTVs respectively. Initial planning and replanning adhered to the same target prescription dose and dose limitation for essential structures.

### Follow-up and data collection

All patients were observed one month after therapy, every three months for the first two years, every six months in the third to fifth years, and annually thereafter. Every subsequent appointment included a flexible fiberoptic endoscopy, head-and-neck scan, abdominal ultrasound, chest X-ray/CT, and blood tests. Electronic medical record system data on medical history, physical examination, blood tests, contrast-enhanced CT and MRI of the head and neck, chest CT, abdominal ultrasound, and bone emission computed tomography scans were obtained. All participants were restaged in accordance with the 8th staging system of the American Joint Committee on Cancer (AJCC). Locoregional failure was identified as a nasopharynx cancer recurrence and/or regional lymph node that was confirmed by biopsy or positron emission tomography/computed tomography (PET/CT). Vital status was determined using follow-up phone calls combined with medical records.

### Failure pattern description

For locoregional recurrent patients, the MRI or CT images acquired at the time of locoregional recurrence were imported on the treatment planning systems. Image fusion of the recurrent scans with the previous simulation CT scans was performed based on bone landmarks. As for patients with replanning, the recurrent scans were integrated with the initial simulation CT scans and the re-simulation CT scans respectively. On the fused images, the recurrent GTVs of the primary site (rGTVnx) and neck lymph nodes (rGTVnd) were delineated layer by layer. The precise location of the recurrent GTVs was then assessed according to the fused images. If the recurrent GTVs were located outside GTVs of the re-simulation CT scans but inside the GTVs of the initial simulation CT scans, the location of the recurrences was defined as “shrunken area”. The type of recurrence was determined based on the 95% isodose lines. The last replans were used for dosimetric analyses in patients with replanning. If 95% of recurrent GTV was inside the 95% isodose, the pattern of recurrence was considered a “in-field” failure. If 20% to 95% of recurrent GTV was within the 95% isodose, the pattern of recurrence was considered a “marginal” failure. If less than 20% of recurrent GTV was inside the 95% isodose, the pattern of recurrence was considered a “out-field” failure [16].

### Statistical analysis

Categorical variables could be represented by frequency and proportion of baseline attributes. Continuous variables could be represented by median and interquartile ranges (IQRs), while discrete variables may be represented by mean and standard deviations (SDs). The statistical analysis was performed using SPSS (IBM, Chicago, IL, version 22.0). Categorical variables were subjected to an X^2^ test. On continuous variables, a paired t-test was conducted to evaluate group variations. Survival rates were calculated from the date of initial treatment to the date of the event or the final follow-up visit. The estimates of survival rates were estimated using Kaplan–Meier method. The significant variation between survival curves were compared using the log-rank test. All tests of statistical significance were conducted using a two-sided distribution, and if the P value of the results was less than 0.05, they were considered significant.

## Results

A total of 274 patients with non-metastatic NPC were involved in this research. There were 174 patients who were undergoing IMRT with adaptive replanning, and 100 patients who were receiving IMRT without replanning. Among the patients with replanning, 123 were male and 51 were female. Among the patients without replanning, 67 were male and 33 were female. Clinical characteristics between the patients with and without replanning appeared well balanced (P > 0.05). More details are displayed in Table 1.

**Table 1:** Patient characteristics

	IMRT without replanning (N = 100), n (%)	IMRT with replanning (N = 174), n (%)	P
Sex			0.524
Male	67 (67.0)	123 (70.7)	
Female	33 (33.0)	51 (29.3)	
Median age (IQR), years	55 (49–65)	56(47–65)	0.776
Smoking			0.317
Yes	40 (40.0)	80 (46.2)	
No	60 (60.0)	93 (53.8)	
T stage			0.782
1	23 (23.0)	39 (22.4)	
2	34 (34.0)	50 (28.7)	
3	24 (24.0)	46 (26.4)	
4	19 (19.0)	39 (22.4)	
N stage			0.752
0	19 (19.0)	29 (16.7)	
1	22 (22.0)	47 (27.0)	
2	52 (52.0)	89 (51.1)	
3	7 (7.0)	9 (5.2)	
Clinical stage			0.998
I	5 (5.0)	9 (5.2)	
II	20 (20.0)	34 (19.5)	
III	50 (50.0)	86 (49.4)	
IVa	25 (25.0)	45 (25.9)	
Chemotherapy			0.934
AC	86 (86.0)	147 (84.5)	
IC	9 (9.0)	18 (10.3)	
No	5 (5.0)	9 (5.2)	

IMRT = Intensity modulated radiation therapy. IQR = Interquartile range. AC = Adjuvant chemotherapy. IC = Induction chemotherapy

The median follow-up time was 67 months (IQR, 35–80). Overall, 45 patients (16.4%) developed distant metastases, and 34 patients (12.4%) developed locoregional recurrences, with 22 occurring at primary site, 10 at the neck lymph nodes, and 2 at both the primary site and the neck nodes. Detailed failure patterns between patients with and without replanning are shown in Table 2.

**Table 2:** Failure patterns of patients treated with IMRT with and without replanning

Patterns of failure	Total (N = 274), n (%)	IMRT without replanning (N = 100), n (%)	IMRT with replanning (N = 174), n (%)
Locoregional recurrence	34 (12.4)	19 (19.0)	15 (8.6)
Local recurrence alone	22 (8.0)	12 (12.0)	10 (5.8)
Regional recurrence alone	10 (3.7)	7 (7.0)	3 (1.7)
Local and regional recurrence	2 (0.7)	0 (0)	2 (1.1)
Distant metastasis	45 (16.4)	17 (17.0)	28 (16.1)
Bone	18 (6.6)	6 (6.0)	12 (6.9)
Lung	19 (6.9)	8 (8.0)	11 (6.3)
Liver	13 (4.7)	4 (4.0)	9 (5.2)
Locoregional recurrence and distant metastasis	4 (1.5)	4 (4.0)	0 (0)

IMRT = Intensity modulated radiation therapy

**Fig. 2:**
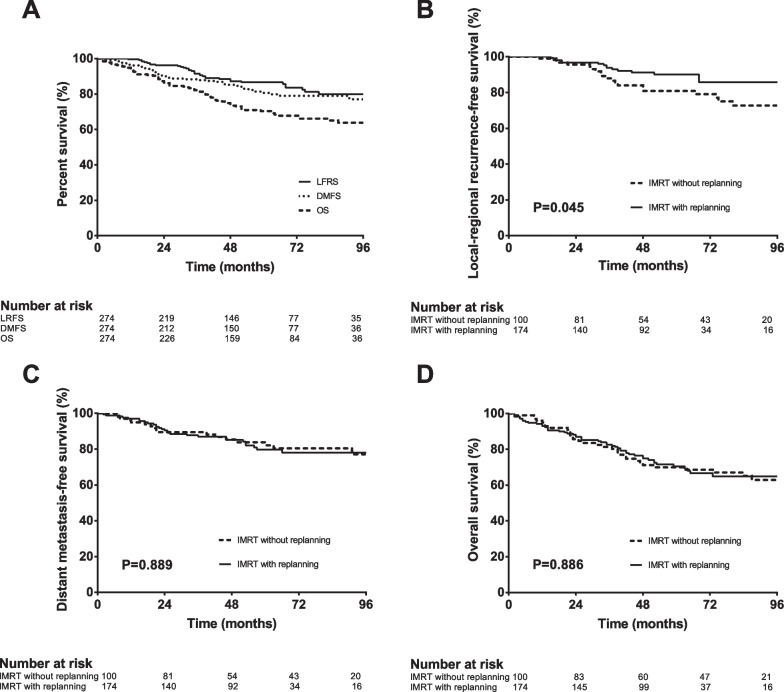
Locoregional recurrence-free survival (LRFS), distant metastasis-free survival (DMFS) and overall survival (OS) for 274 patients with non-metastatic NPC (**A**); LRFS (**B**), DMFS (**C**), and OS (**D**) for 174 NPC patients received IMRT with replanning and 100 patients without replanning

Replanning throughout IMRT for non-metastatic NPC significant increased LRFS, but neither DMFS nor OS (Fig. 2). The 5-year LRFS, DMFS, and OS rates were respectively 86.7%, 80.6%, and 70.4%. Comparing patients with and without replanning, the 5-year LRFS rates were 90.1% (95%CI, 84.8% to 95.4%) and 80.8% (95%CI, 72.0% to 89.6%), respectively (P = 0.045). The 5-year DMFS rates for patients with and without replanning were 79.7% (95%CI, 72.6% to 86.8%) and 82.1% (95%CI, 73.7% to 90.5%), respectively (P = 0.889). The 5-year OS rates for patients with and without replanning were 70.5% (95%CI, 63.1% to 77.9%) vs 69.9% (95%CI, 60.5% to 79.3%), respectively (P = 0.886).

Table 3 displayed the full DVH data of IMRT planning for patients with local–regional recurrence. In general, both patients with and without replanning had excellent dose coverage of the target volumes. The percentage of target volumes receiving 100% of the prescribed dose (V100) were all more than 95%. There were no significant differences between patients who received IMRT with and without replanning.

**Table 3:** DVH statistics for patients of recurrence

	IMRT without replanning	IMRT with replanning	P
GTVnx			
Dmean (cGy)	73.19 ± 2.18	72.48 ± 1.43	0.260
Dmax (cGy)	76.63 ± 2.44	76.32 ± 1.92	0.693
Dmin (cGy)	66.73 ± 2.27	65.43 ± 2.94	0.155
V100%	97.4 ± 3.0	97.6 ± 2.0	0.864
GTVnd			
Dmean (cGy)	72.75 ± 0.67	72.58 ± 0.60	0.489
Dmax (cGy)	75.72 ± 1.13	75.79 ± 1.38	0.891
Dmin (cGy)	68.88 ± 1.61	69.02 ± 1..34	0.803
V100%	99.3 ± 0.7	99.6 ± 0.7	0.330
CTV1			
Dmean (cGy)	66.48 ± 2.10	66.80 ± 1.68	0.629
Dmax (cGy)	77.25 ± 1.89	76.86 ± 1.63	0.526
Dmin (cGy)	49.94 ± 3.68	49.00 ± 2.79	0.416
V100%	98.1 ± 1.0	98.4 ± 0.6	0.278
CTV2			
Dmean (cGy)	56.89 ± 1.47	57.32 ± 1.06	0.414
Dmax (cGy)	62.65 ± 1.89	62.93 ± 1.09	0.657
Dmin (cGy)	48.37 ± 9.43	52.30 ± 1.02	0.184
V100%	96.5 ± 2.3	97.5 ± 1.7	0.226

DVH = Dose-volume histogram. IMRT = Intensity modulated radiation therapy. GTVnx = Gross tumor volume of primary tumor. GTVnd = Gross tumor volume of neck lymph nodes. CTV1 = Clinical tumor volume of the high-risk region. CTV2 = Clinical tumor volume of the low-risk region. Dmean = Mean dose. Dmax = Maximum dose. Dmin = Minimum dose. V100% = The percentage of the target volume covered by the 100% prescribed dose line

All 34 locoregional recurrent patients with 36 recurrent sites were under analysis. Except for one (5.9%) out-field failure at primary site, all recurrent patterns (94.1%) were considered to be in-field failures in patients who received IMRT with replanning. There was no recurrence occurring at the shrunken area after adaptive replanning during IMRT, 13 of 17 (76.5%) locoregional recurrences located inside replanning GTVs, 3 of 17 (17.6%) located inside replanning CTVs, and 1 (5.9%) located outside replanning GTVnx. Among patients without replanning, sixteen patients (84.2%) consisting of 10 local recurrences and 6 regional recurrences were considered in-field failures, two patients (10.5%) consisting of one local recurrence and one regional recurrence were considered to be marginal, one patient (5.3%) with local recurrence was considered to be out-field failure. The details of recurrent patients and their failure patterns are shown in Table 4 (patients with replanning) and Additional file 1: Table S1 (patients without replanning).

**Table 4:** Details of recurrent patients receiving IMRT with replanning and their failure patterns

No.	Sex	Age (y)	Stage	Site of recurrence	Location of recurrence	V95%	Type of recurrence
43	Male	44	T3N0	Local	GTVnx	97.7	In-field
57	Male	50	T1N0	Local	GTVnx	96.1	In-field
61	Female	34	T3N2	Local	GTVnx	95.9	In-field
96	Male	50	T3N2	Local	Outside GTVnx	10.01	Out-field
				Regional	CTV2	100.0	In-field
100	Male	60	T3N2	Local	GTVnx	97.1	In-filed
130	Female	72	T4N0	Local	GTVnx	97.5	In-filed
162	Male	59	T3N1	Local	GTVnx	99.8	In-filed
186	Male	47	T2N1	Regional	CTV1	98.68	In-field
191	Male	37	T2N2	Local	GTVnx	96.8	In-field
				Regional	CTV1	100.0	In-field
194	Male	55	T4N0	Local	GTVnx	99.2	In-field
200	Male	49	T4N2	Local	GTVnx	96.8	In-field
202	Female	43	T2N2	Regional	GTVnd	99.3	In-field
208	Female	72	T4N3	Local	GTVnx	97.1	In-field
214	Male	60	T4N2	Local	GTVnx	99.5	In-field
256	Male	66	T2N2	Regional	GTVnd	100.0	In-field

IMRT = Intensity modulated radiation therapy. GTVnx = Gross tumor volume of primary tumor. GTVnd = Gross tumor volume of neck lymph nodes. CTV1 = Clinical tumor volume of the high-risk region. CTV2 = Clinical tumor volume of the low-risk region. V95% = The percentage of the target volume covered by the 95% prescribed dose line

## Discussion

In the era of IMRT, the advantages of ART in ensuring adequate dose for target volumes and safe dose for essential normal structures have led to its widespread use in the treatment of NPC [17]. Previous studies focused on the benefits of adaptive replanning in treatment of NPC which included increase LRFS rate, alleviate side effects, and improve QoL [10, 11, 15, 18]. Moreover, information on contouring the target volumes of replanning for optimizing the early response during IMRT in NPC is limited. No conclusions have yet been reached on how to describe the target volumes and the appropriate radiation dose of adaptive replanning. The current study demonstrates the feasibility of adapting the target volumes and reducing the doses to the area of tumor reduction throughout the course of IMRT, and presents our experiences regarding the description of target volumes for adaptive replanning and the optimal dose for area of tumor reduction in NPC.

Owing to the fundamental principles of radiobiology [19], only a large tumor burden necessitates a higher radiation dose for effective treatment. Previous research had shown a radiation dose of 50 Gy was effective for controlling over 90% of subclinical diseases, 60 Gy for controlling microscopic diseases, and a higher dose for treating clinically identifiable diseases in NPC [20]. However, for the primary tumor and nodal mass shrunk to subclinical lesions during IMRT, dosing with the same amount of radiation as the primary tumor seems unreasonable. Consequently, we hypothesized that the residual diseases that could be seen by CT or MRI during IMRT still had a significant tumor burden and must be distinguished as GTVs obtaining the adequate radiation dose. Regarding tumor shrinkage in which there was a dramatic drop in tumor cell count and undetectable by CT or MRI, the disease might be delineated into the high-risk region getting a relatively lower dose, such as 60 Gy. This therapeutic strategy could not only be conducive to give the adequate dose to the residual disease and tumor shrunken area during IMRT, it is also necessary to further decrease the high-dose region of organs at risk, especially for those who had large tumor closed to or even overlapped critical normal structures. Similar approaches were used to describe the target volumes and appropriate dose for tumor shrinkage following induction chemotherapy in NPC patients [21–24].

There is limited study concerning the description of target volumes and appropriate dose for tumor shrunken area in patients with NPC receiving adaptive replanning during IMRT. Hansen et al. maintained the size of the initial GTVs when recontoured the GTVs for replanning [25]. Zhao et al. recontoured GTVs based to the shrinkage and deformation of the primary tumor and lymph nodes shown by re-simulated CT imaging, while maintaining the size of the initial CTVs. Excellent local–regional control was established in their research group, with a 3-year local relapse-free survival rate of 72.71% for patients receiving replanning. Additionally, the radiotherapy related acute and late toxicities were alleviated [10]. However, the failure patterns especially for locoregional recurrences were not analyzed, which was relatively important for evaluating the feasibility of this specific strategy. Xie et al. conducted a study based on 54 NPC patients receiving IMRT with adaptive replanning. They defined replanning GTVs as all residual diseases, replanning CTV1 as the same as the initial CTV1, and replanning CTV2 was not delineated. Over 65 Gy was administered to the tumor regression area, and a total of 45–47 Gy was given for CTV2 over the course of 25–26 fractions. After a median follow-up time of 30 months, four patients developed locoregional recurrence with none occurring in the area of regression or CTV2 area [26].

In the present study, 274 participants with non-metastatic NPC who had IMRT with and without replanning were evaluated. Tumor regression area was included in replanning CTV1 instead of replanning GTVs, and the prescribed dose for this area was 60–66 Gy. Consistent with previous reported [10, 11, 15], IMRT with replanning could significantly improve the local regional control for NPC patients. Among 100 patients without replanning, 19 patients had locoregional recurrences, 16 (84.2%) were considered as in-field failure, 2 (10.5%) were considered as marginal failure, and 1 (5.3%) was out-field failure. Among 174 patients with replanning, 15 patients had 17 locoregional recurrences, 16 (94.1%) were considered as in-field failure, and 1 (5.9%) was out-field failure. No marginal recurrence was observed in patients with replanning, which means that reducing the GTVs and the radiation dose prescribed for tumor shrunken area does not produce additional locoregional recurrence in this area. Our finding indicated that adapting the target volumes and altering the radiation dose prescribed to the area of tumor reduction were achievable. It should be noted that in the current research, for the bony structures of skull base invasion, the target volumes were described depending on the initial images despite tumor regression during the course of IMRT.

The parotid gland is one of the most vulnerable organs during IMRT for head and neck (H&N) tumors, excessive dose to the parotid glands results in the increased risk of toxicities, such as xerostomia, dysphagia, and dependence on nasogastric tube feeding [27]. Anatomical changes and shrinkages of the parotid glands are common during RT for H&N tumors [28], and these variations can usually result in overdosed to the organ at risk. The use of adaptive replanning, taking into consideration the change in anatomical changes and volume modifications, could be useful in reducing dose to the organ at risk, such as parotid glands [29]. Consistent with the most studies in H&N tumors, we previously reported that adaptive replanning could reduce the mean dose of the parotid glands up to 4.23Gy [7]. It was expected that the risk of requiring reactive enteral feeding through a nasogastric tube would reduce considerably according to a specific predictive model [30].

It is important to acknowledge that, though ART has been well described for decades, the routine application of CT-based ART remains relatively limited. One of the most important reasons is that CT usually cannot provide sufficient soft tissue contrast to accurately identify normal structures and tumors. However, MRI can provide relatively higher soft tissue contrast and superior target volume delineation than CT. Recently, the introduction of MR-linac provides a helpful technology for ART. This technology is of great interest in abdominal, pelvic and H&N tumors. Preliminary results demonstrated that MR-guided ART was feasible and well tolerated with minimal toxicity and encouraging tumor outcomes [31]. Another emerging technology worth mentioning is proton beam therapy (PBT), which has gained increasing interest for its advantage to perform even more conformal dose distribution with better sparing of surrounding normal structures [32]. The application of PBT in the treatment of H&N tumors has been growing in the past few years and has shown its potential association with reduced toxicity burden [33, 34]. A phase 3 randomized clinical trial assessing whether PBT reduces toxicity in oropharyngeal cancer (TORPEDO trial) [35] has recent completed accrual, and will give an answer soon on the potential role of PBT in this setting. It is expected that adaptive replanning with PBT could further improve its favorable dose distribution and reduce toxicities. Moreover, PBT has become more widely accessible over the past few years and expansion of commissioning to include these indications is anticipated [32].

There are several limitations that should be addressed in our study. Although all of the patients in the current cohort had popular treatment modalities at that time, the treatment modalities were not completely identical along the time frame especially for the systemic treatment strategies. Most of the patients in this study received adjuvant chemotherapy instead of induction chemotherapy. Since the patients receiving induction chemotherapy might have limited response to 1st IMRT fractions, the percentage change in volume for replanning PGTV in comparison to baseline PGTV might be small. Thus, the findings need to be explicated thoroughly and confirmed by elaborately conducted investigations in the future. Another limitation of the present study is the lack of information regarding toxicity. Although this issue is not in the scope of the present study, we have to acknowledge that, the aim of ART is the reduction of toxicities without jeopardizing tumor control. A follow up study is on the way to address this issue.

## Conclusions

In conclusion, in-field failure inside the high dose area was the most common locoregional recurrent pattern for non-metastatic NPC. Adapting the target volumes and modifying the radiation dose prescribed to the area of tumor reduction during IMRT with adaptive replanning were feasible and would not detrimental for locoregional tumor control.

### Supplementary Information

**Additional file 1. Table S1.** Details of recurrent patients receiving IMRT without replanning and their failure patterns.

## Data Availability

The data that support the findings of this study are available on reasonable request from the corresponding author.
