# Supplementary material for: Failure patterns of locoregional recurrence after reducing target volumes in patients with nasopharyngeal carcinoma receiving adaptive replanning during intensity-modulated radiotherapy: a single-center experience in China
Source: Radiat Oncol. 2023 Nov 16;18:190. doi: 10.1186/s13014-023-02373-7 (PMC10652536; doi:10.1186/s13014-023-02373-7)
Supplement: Supplementary file 1 — Additional file 1. Table S1. Details of recurrent patients receiving IMRT without replanning and their failure patterns. [file 13014_2023_2373_MOESM1_ESM.docx]

| Supplement Table 1. Details of recurrent patients receiving IMRT without replanning and their failure patterns | | | | | | | |
| --- | --- | --- | --- | --- | --- | --- | --- |
| No. | Sex | Age (y) | Stage | Site of recurrence | Location of recurrence | V95% | Type of recurrence |
| 22 | Male | 51 | T2N3 | Local | GTVnx | 97.5 | In-field |
| 37 | Male | 42 | T3N1 | Local | GTVnx | 96.9 | In-field |
| 46 | Male | 68 | T4N2 | Regional | CTV1 | 98.1 | In-field |
| 52 | Female | 58 | T4N2 | Regional | CTV1 | 100.0 | In-field |
| 59 | Male | 40 | T3N2 | Local | GTVnx | 99.2 | In-field |
| 72 | Female | 55 | T2N2 | Local | Outside GTVnx | 11.1 | Out-field |
| 75 | Female | 48 | T3N1 | Regional | CTV1 | 96.5 | In-field |
| 81 | Male | 68 | T3N2 | Local | GTVnx | 99.4 | In-field |
| 90 | Female | 43 | T2N1 | Local | GTVnx | 98.1 | In-field |
| 101 | Male | 57 | T4N2 | Local | GTVnx | 98.66 | In-field |
| 104 | Male | 51 | T2N1 | Local | GTVnx | 100.0 | In-field |
| 109 | Female | 54 | T4N0 | Local | Marginal to GTVnx | 36.0 | Marginal |
| 110 | Male | 69 | T3N2 | Regional | CTV1 | 100.0 | In-field |
| 148 | Male | 54 | T3N2 | Local | GTVnx | 96.2 | In-field |
| 160 | Male | 57 | T1N2 | Regional | GTVnd | 99.91 | In-field |
| 168 | Male | 57 | T1N2 | Regional | CTV1 | 98.12 | In-field |
| 187 | Male | 57 | T2N2 | Local | GTVnx | 100.0 | In-field |
| 252 | Male | 37 | T3N2 | Local | GTVnx | 100.0 | In-field |
| 261 | Male | 59 | T2N2 | Regional | Marginal to CTV1 | 26.8 | Marginal |
| Abbreviations: IMRT=Intensity modulated radiation therapy. GTVnx=Gross tumor volume of primary tumor. GTVnd=Gross tumor volume of neck lymph nodes. CTV1=Clinical tumor volume of the high-risk region. V95%=The percentage of the target volume covered by the 95% prescribed dose line. | | | | | | | |
